# Love vs. Risk: Women with Sickle Cell Disease Face Reproductive Decision-Making Dilemmas

**DOI:** 10.3390/ijerph22030342

**Published:** 2025-02-26

**Authors:** Lisa R. Roberts, Carlene O. Fider, Safiye Sahin, Jayde Frederick, Ilsa Nation, Susanne Montgomery

**Affiliations:** 1 School of Nursing, Loma Linda University, Loma Linda, CA 92354, USA; cfider@llu.edu (C.O.F.); ssahin@llu.edu (S.S.); 2 School of Medicine, Loma Linda University, Loma Linda, CA 92350, USA; jfrederick@students.llu.edu; 3 Sickle Cell Services, Loma Linda University Medical Center, Loma Linda, CA 92354, USA; ination@llu.edu; 4 School of Behavioral Health, Loma Linda University, Loma Linda, CA 93350, USA; smontgomery@llu.edu

**Keywords:** mental health, reproductive health, sickle cell anemia, social determinants of health

## Abstract

Sickle cell disease/trait (SCD/T) is the most common genetic blood disorder in the U.S., characterized by painful vaso-occlusive crises resulting in considerable morbidity and premature death. Advances in treatment have somewhat improved the quality of life and longevity. Therefore, people with SCD/T are now living into their reproductive years. However, pregnant individuals with SCD have a maternal mortality risk of up to 26 times higher than the national average. Individuals with sickle cell trait also have an increased risk of untoward maternal health outcomes. We sought to understand reproductive health concerns among women with SCD/T, through data collected from patients, caregivers, advocates, and healthcare professionals using key informant interviews and focus groups (*N* = 54). Audio recordings were transcribed verbatim, coded inductively, and analyzed thematically. Three major themes emerged: (1) the Dilemma of Love vs. Risk, (2) SCD/T Knowledge, and (3) the Mental and Emotional Toll of SCD/T. Reproductive concerns and experiences among women with SCD/T influence their mental health and social engagement. Programs are urgently needed that address the unique SCD/T reproductive health risks and communication and support needs. These include readily accessible, age-appropriate SCD/T reproductive health information, counseling, and engaging communication tools for women and their potential partners. Support requires a multidisciplinary approach.

## 1. Introduction

Sickle cell disease/trait (SCD/T) is the most common genetic blood disorder in the United States (US), with SCD affecting approximately 100,000 individuals and nearly 3,000,000 having the trait [1,2]. SCD is a genetic disorder that affects hemoglobin in red blood cells, leading to various health complications. In the US, African Americans/Blacks are most affected [3], which adds to the complexity of the illness. With a primary complication of pain, misunderstandings about disease management are often conflated by stereotypes and prejudice [4]. While persons affected by SCD/T face many health challenges, one that is rarely talked about is disease presentations when women get pregnant. Indeed, the lack of reproductive health knowledge significantly impacts individuals with sickle cell disease (SCD), particularly women. For women with SCD, reproductive health issues are complex and, as a result, often under-addressed. Many women with SCD face challenges related to menstruation, contraception, fertility, and pregnancy [5].

Pregnant people with SCD have a risk of maternal mortality that is 26 times higher than average [6]. As can be seen in the Table 1, they also face higher risks of adverse maternal and perinatal outcomes, such as an increased risk of cardiovascular disorders including cardiomyopathy, stroke, venous thromboembolism, various infections, and poor fetal growth [6,7,8]. Additionally, the risk of stillbirth was found to be more than doubled among Black pregnant women with SCD in California, and they had 2.25 times the risk of postpartum hemorrhage, compared to Black women without SCD [9]. A recent US population study indicated that women with SCD had an increased relative risk (RR) of between 3.70 and 10.90 for SCD-specific complications compared to women without SCD or other anemia—for instance, an RR of 4.99 for acute respiratory distress syndrome, which included acute chest syndrome [8]. Individuals with sickle cell trait also have an increased risk of adverse maternal health outcomes, including preeclampsia, preterm delivery, and stillbirth [10]. Notably, contrary to popular misunderstandings and prejudice, women in these clinical studies presented with no risk for substance abuse (OR 1.0).

Women with sickle cell disease (SCD) or sickle cell trait (SCT) often face unique reproductive concerns that can significantly impact their mental health. The risk of passing the disease to their offspring, coupled with the potential complications during pregnancy, can lead to heightened anxiety and stress. Studies have shown that women with SCD are at an increased risk of severe maternal morbidity and mortality which adds to their reproductive concerns [11]. This constant worry about their health and their child’s health can contribute to chronic stress and mental health issues such as depression and anxiety [2].

Moreover, the decision-making process regarding reproductive choices can be fraught with emotional turmoil. For example, higher rates of postpartum sterilization among women with SCD raise questions about reproductive autonomy and the psychological impact of such decisions [12]. The physical symptoms of SCD, such as pain crises and fatigue, are aggravated during pregnancy and can interfere with daily life and contribute to feelings of helplessness and frustration. Pregnancy is also a time when women must consider changing their pain management. These physical and emotional burdens can create a cycle of stress and mental health deterioration.

Effective communication plays a pivotal role in addressing challenges related to SCD and fostering both mental health and relationship growth [13]. Open and informed discussions between individuals with SCD and their partners are particularly crucial, as these conversations help navigate the complexities of reproductive health decision-making and the toll of living with the disease. Communication allows partners to share fears, anxieties, and expectations, fostering mutual understanding and emotional intimacy. This openness is essential in managing stressors such as decisions about family planning, potential genetic risks, and the unpredictability of health complications. In the context of intimate relationships, effective communication strengthens trust and collaboration. The current research therefore sought to understand the intersection of reproductive life events with mental health for women with SCD.

This study aimed to explore reproductive health concerns among women with SCD/T by gathering insights from patients, caregivers, advocates, and healthcare professionals. Our research question was the following: How do women with SCD/T experience and manage reproductive decision-making?

## 2. Materials and Methods

We developed our theoretical model from a previously published Life Course Theory diagram of adverse childbearing outcomes [14] and essential components of the Integrated Behavioral Model, which expands on the theories of Reasoned Action and Planned Behavior [15].

Using a qualitative research approach and purposive triangulated sampling to ensure the inclusion of relevant experiences and perspectives [16], data were collected from patients with SCD/T, caregivers, advocates, and healthcare professionals. Participants were recruited through clinics, community organizations, and health fairs, with the inclusion criteria requiring individuals to be aged between 12 and 25 years and have a diagnosis of SCD/T (for patient participants) or be directly involved in SCD/T care or advocacy (both caregivers and healthcare providers). Individuals unable to provide informed consent or those without direct experience of SCD/T were excluded. Using a semi-structured guide with open-ended questions and follow-up probes aligned with our theoretical model, we explored participants’ perspectives regarding how having SCD/T affects daily living; communication and dating while having a chronic, complex illness; family planning knowledge, concerns, and experiences; and coping strategies.

Data were collected in San Bernardino county, which has the second-largest population of people with SCD/T in California [17]. The inquiry began initially through an adult patient needs and assets assessment, which was expanded as we learned more about the needs of young adults, including dating and family planning, which led to further exploration of related issues through in-depth key informant interviews and two validation focus groups (*N* = 54). Audio recordings were transcribed verbatim, coded inductively, and analyzed thematically [18]. Thematic analysis was chosen because it provides a structured yet flexible approach for identifying patterns across diverse participant experiences [19]. This method aligned well with our theoretical framework, allowing us to capture both individual and shared perspectives on SCD/T while maintaining adaptability to emerging themes. Compared to a grounded theory or content analysis, thematic analysis was particularly well suited for synthesizing complex, multifaceted data without requiring the development of a new theoretical model [20].

All interviews were conducted in English, after obtaining informed consent. The authors’ Institutional Review Board (IRB) approved the studies (IRB #5190109, 28 December 2021; #5230484, 30 October 2023).

## 3. Results

The participants were primarily African American and some African immigrants with SCD/T (*n* = 22). Most (85.8%) knew their SCD type or trait status. While 42.9% of the participants reported that they were in a current dating relationship, the rest reported they were not. Other participants included (*n* = 16) caregivers, and the remaining participants (*n* = 16) were healthcare providers, support staff, and administrators.

**Figure 1:**
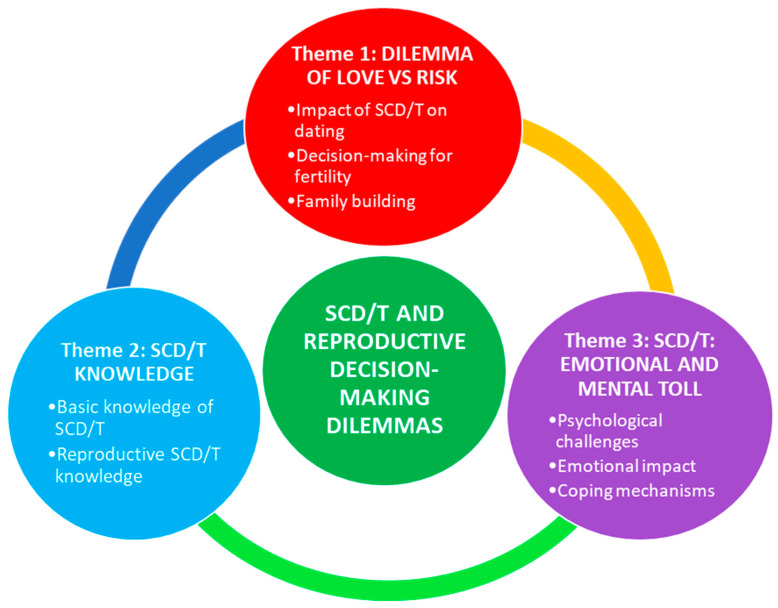
Women’s overarching dilemmas with themes and subthemes.

Figure 1 shows the female participants’ overarching dilemmas with major themes and subthemes. Three major themes emerged with the coding and analysis of the transcripts, which convey separate but at times overlapping issues pertaining to the influence of reproductive health concerns on mental health among women with SCD/T. The first theme, the *Dilemma of Love* vs. *Risk*, expresses the impact SCD/T has on dating, decision-making around fertility, and family building choices in the voices of the women affected. The second theme, *SCD/T Knowledge*, provides examples of how basic SCD/T knowledge and an understanding of genetic risks and reproductive health, or a lack thereof, plays a role in their romantic relationships. The third theme, the *Mental and Emotional Toll of SCD/T*, explicates the psychological challenges and emotional impact of living with a chronic, complex disease that has implications for their choice of romantic partners and coping mechanisms employed to deal with the entire dilemma of love vs. reproductive health risks associated with dating as a person with SCD/T.

### 3.1. Dilemma of Love vs. Risk

**Figure 2:**
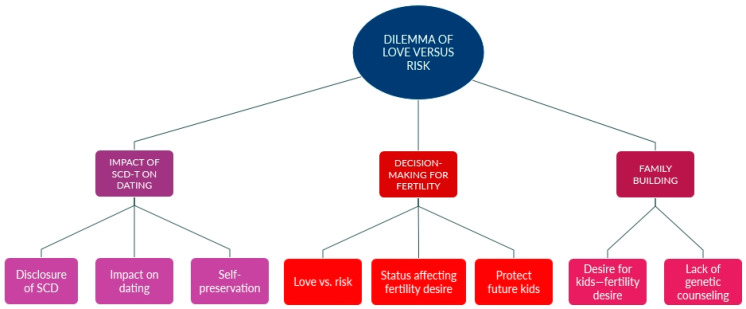
Dilemma of Love vs. Risk, with subthemes and codes.

Figure 2 shows the subthemes and codes for the theme of the “Dilemma of Love vs. Risk”.

The impact of SCD/T on dating, romantic relationships, and marriage was palpable as participants expressed constant uncertainty regarding their reproductive future. The unpredictable nature of SCD—“I’m concerned if I’m going to be able to conceive or not…my health is so different. You never know year by year.” (Participant #17, female)— combined with a lack of support, makes dating and reproductive health decision-making difficult: “I finally got pregnant and he [the hematologist] was like, I’ll see you in nine months.” (Focus Group Participant A). Participants also expressed uncertainty due to a lack of genetic counseling to more fully understand the information made available to them. While desiring to have children, or at least the opportunity to do so in the future, this was overshadowed by wanting to protect their offspring from the terrible suffering of inheriting SCD/T. Some participants, not certain if this was even possible, would choose to forego romantic relationships and having children rather than risk it.

*“My last partner, yes. And I mean, we’re both super young, so we’re like, ‘Let’s hold on that.’ And if it does happen to, like we’ll adopt someone that’s a child that’s a couple months so we could like raise them.”* (Participant #19, female).

*“Would I take the risk for love and potentially put my child in literal pain, or walk away from that [relationship]?”* (Participant #13, female).

*“I’m on hydroxyurea, and I know that, while you’re on hydroxyurea, you can’t be pregnant, or it will hurt the baby.”* (Participant #17, female).

These quotes reflect the dilemma that participants expressed—the terrible feeling of having to choose between managing the pain related to their illness and their partner’s desire to have a child, as well as the love for future offspring being overshadowed by worry regarding their risk of inheriting the illness. In effect, they were constantly weighing the risk to their own health, as well as that of their children, with an age-appropriate human desire to have a family.

Healthcare providers aware of the risks found it difficult to navigate the topic with patients. Best practices such as pre-conception genetic counseling and shared decision-making are essential, yet may not occur in a timely manner or in a manner conducive to partnering with patients as they navigate this difficult dilemma.

*“There is a very high incidence of maternal and fetal morbidity in people with sickle cell disease. So … planning fertility and, reproductive outcomes is extremely important and critical.”* (Participant #10, HCP).

*“I looked at my ex-husband and I said, “What do you want to do?” And he said, “I don’t want an abortion.” And I said, I didn’t either. And I told the counselor and they were adamant, ‘You have to terminate.’ And I was like, this is my body, this is my child, I am not terminating my child.”* (Participant #4, caregiver).

*“So then we had our second son, and the same thing. They told us that he had the trait.”* (Participant #3, caregiver).

*“… some of them have lost multiple [pregnancies], they’ve gone through multiple fetal demises, and it’s not healthy that they go through that often.”* (Participant #6, caregiver).

These are difficult conversations for patients to have with their romantic partners, as well as with healthcare providers. Such conversations are fraught with conflicting emotions, issues of trust, and the need for timely, accurate information, and for most, these did not start at young enough ages to have had sufficient time to wrestle with the issues. Rather, these conversations tend to occur at a time when they are already pregnant or are seriously considering starting a family.

### 3.2. SCD/T Knowledge

**Figure 3:**
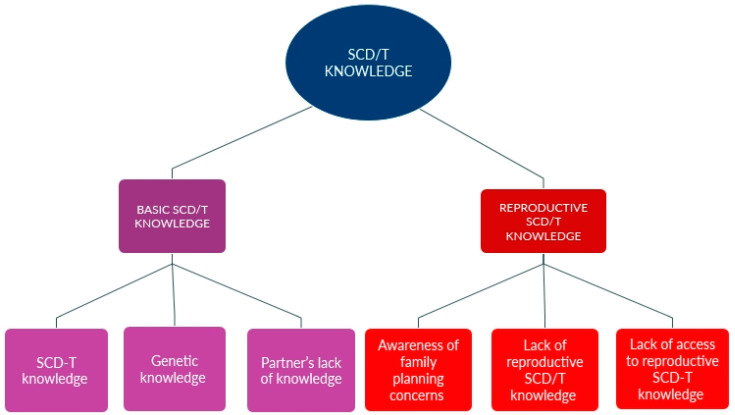
SCD/T Knowledge subthemes and codes.

The theme of SCD/T Knowledge is illustrated in Figure 3. Most participants, while having a rudimentary understanding of SCD/T, were less informed regarding SCD/T-specific reproductive health and expressed difficulty finding access to the needed information.

*“To have kids? I don’t think it’s that risky, because they say that sickle cell patients can’t have kids, but my cousins had kids, my best friend’s mom, she had three kids.”* (Participant #20, female).

*“For almost … their entire pregnancy, and then they come out, and they are diagnosed with something, and they are crying. So, it’s lack of knowledge and education….”* (Participant #6, caregiver).

The lack of engaging educational tools regarding parental planning and maternal health while having sickle cell, as well as how to communicate with romantic partners, was noted repeatedly. Patients reported feeling unprepared and, therefore, often avoided difficult conversations about their SCD/T status. “It would be good to know how to have that conversation with someone about your reproductive health.” (Participant #17, female). Having a partner who lacked an understanding of SCD/T and the genetic implications was also expressed as a concern. “No, I think he tried to understand. Yeah, he did his own research, which I appreciated, but I still don’t think he understood the depth of it *…* I think they thought it was more something like asthma.” (Participant #17, female). Even patients who were well informed about their own health had difficulty initiating the conversation with potential partners or even current partners and felt unprepared to educate them about the disease and their reproductive health concerns.

Early in relationships, participants struggled with when, how, and how much to disclose about having SCD/T. “Because I think that’s probably the biggest part, and definitely the biggest hurdle, that either you can choose to really be in the uncomfortable and engage with that sort of conversation…” (Participant #13, female). Participants varied in their perspectives of when to disclose this, from disclosing their SCD/T status before establishing a relationship to keeping the information to themselves despite an ongoing relationship. “I would just tell him I was sick in the hospital *…*. Or sometimes I would lie. I was like, ‘Oh, it was a stomach bug.’ Because he knew my friends, so I didn’t want--my friends didn’t know either.” (Participant #18, female). Participants struggled to maintain a sense of privacy with the need for communication to support healthy relationships. Many felt that disclosing this part of their lives would even undermine the formation of a committed relationship and decided not to share their illness and definitely not to share the implication of the risk to themselves, the complications of a potential pregnancy, and the risk to their unborn child. The idea that they may have to make choices to discontinue life-improving medication if pregnant, which could result in months of higher pain, without having their caregivers discuss options around this can be overwhelming and having providers outright suggest terminations and sterilization is shocking, leading further into a narrative and fears that people of color know all too well.

### 3.3. Mental and Emotional Toll of SCD/T

**Figure 4:**
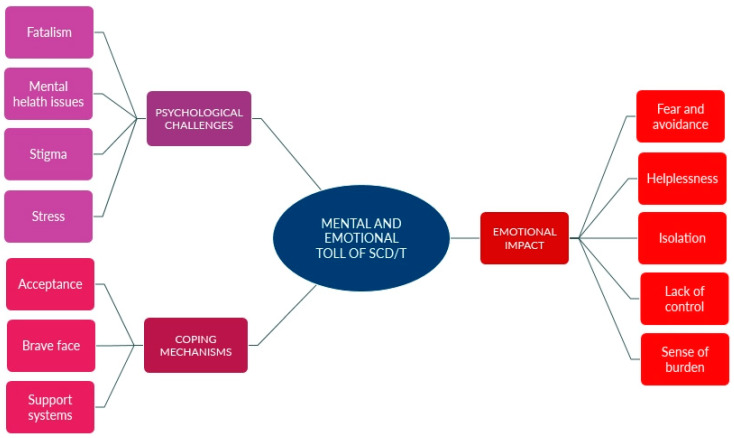
Mental and Emotional Toll of SCD/T, with subthemes and codes.

Figure 4 depicts the theme that emerged of the Mental and Emotional Toll of SCD/T.

The psychosocial challenges of having SCD/T while trying to navigate young adult life, let alone dating, are immense. While knowledge can be empowering for informed decision-making and planning purposes, it does not negate the emotional toll and wearing effect that having to think about SCD/T and make such difficult choices can have on mental health. Participants noted mental health issues such as depression, stress, stigma, and fatalism.

*“Sickle cell is not just about the pain. It’s mentally, it’s emotionally. It’s the jobs that I was talking to you about. It’s about college, school, work. Because it’s like you can have a crisis, you can fall behind in school. Then you start to feel less of yourself and feel like you’re not smart enough. And the same with jobs. So I feel like it can really take a toll on your mental and your emotional state if you allow it to.”* (Participant #8, female).

The emotional impacts experienced by participants included isolation, helplessness, feeling a lack of control, as well as a sense of burden, and fear leading to avoidance behaviors. The mental and emotional toll of having SCD/T is experienced from a young age and carries over to young adulthood.

*“I have one friend who was saying that she doesn’t know why I come with them places because I couldn’t walk as fast as them …. That’s why I decided to just stay at home and not do a lot of things with other people unless they understand.”* (Participant #45).

*“I don’t like for other people to see me in pain like that, or crying, or super drained, and fatigued, so I think that’s something that I just kind of kept to myself, for sure, too.”* (Participant #17, female).

Accumulated incidents of illness and pain crises add layers of isolation and interfere in relationships with peers, partners, and family members. When people with SCD/T feel like they have to put on a brave face, concealing their pain and distress from those they should be able to lean on and confide in, it makes living with a chronic, complex disease even harder.

*“I don’t really have friends like that, a lot of them don’t understand.”* (Participant #20, female).

*“Yeah, I don’t want them to see me as just sick, like all you see is my sickle cell. You don’t see the other side of me.”* (Participant #17, female).

*“So I always show like the strong face, like I’m okay.”* (Participant #19, female).

Energy is spent concealing distress instead of dealing with it. Even seemingly small matters, such as talking with a friend about an interest in a potential partner become heavy with what ifs under the circumstances.

*“Like, stressing about having—whether I get sick or not.”* (Participant #20, female).

*“So I just feel like it’s more than just the disease itself. It opens a door for so much more [difficulties].”* (Participant #14, female).

Caregivers, too, feel the need to conceal the SCD/T status of their loved ones as they hear others refer to persons with SCD/T in derogatory ways and feel the need to protect them by not sharing their secret. Patients and caregivers are very much aware of and sensitive to the stigma surrounding SCD/T within the community and in the healthcare environment. This comes with a high price for both.

*“I’ll say my friends that have kids with full-blown sickle cell, they too don’t--they don’t want to share. They don’t like to talk about it. They’ll tell me things in confidence and let it be like that. It’s not something that you want to say publicly.”* (Participant #3, caregiver).

*“I’ve heard words like ‘This isn’t someone coming in with a congenital condition.’ I hear ‘This is a sickler coming in with a new medical complaint.’ On the sign-outs, there are different words, like ‘sickler,’ like ‘pain seeker,’ like ‘needing opioid medication’ that are discussed.”* (Participant #11, HCP).

Caregivers, advocates, and healthcare providers are aware of the need for mental health services and/or support groups, as with other chronic diseases. One participant suggested [peer support], “*… like a buddy system or something to teach them [young people] how to deal with this lifelong issue.*” (Caregiver Focus Group Participant 4). Participants noted that coping is facilitated by support from their partners: *“Yeah, he understands. He’s always making sure I’m okay whenever we’re around each other.”* (Participant #20, female). Others expressed that to some degree, having an attitude of acceptance is a helpful coping strategy. “*And as I’ve grown I’ve learned to accept it. I feel like when I was younger it was harder because it’s not like I have a normal lifestyle like everyone else.*” (Participant #19, female).

## 4. Discussion

This study was conducted in San Bernardino county, which has the second-largest population of people with SCD/T in California [17]. Data analysis revealed three major themes that highlight the complex interplay between reproductive health concerns and mental health among women with SCD/T. These themes underscore the multifaceted challenges faced by these women in their personal and romantic lives.

### 4.1. Discussion on the Dilemma of Love vs. Risk

The first theme, the “Dilemma of Love vs. Risk”, captures the profound impact of SCD/T on dating, fertility decisions, and family building choices. Previous studies have noted the lack of family planning information available to young people with SCD and that family planning decision-making is influenced by their partner and/or and the opinions of healthcare providers (HCPs) [17,18]. Women with SCD/T often find themselves at a crossroads, balancing their age-appropriate, “normal” desire for romantic relationships and family formation against the risks posed by their condition. The narratives of the participants vividly illustrate this struggle. This uncertainty, coupled with a lack of support from their providers (and at times even actively dismissive attitudes) about how they would even consider this under their circumstances, complicates their decision-making processes. The accounts from the participants reflect the ongoing tension between the desire for love and the fear of potential health risks. In the current research, participants expressed fear of SCD complications and pregnancy complications, as well as worrying about poor maternal/fetal outcomes, which is similar to the findings in prior research [17].

Clearly, the issue of family formation for persons with SCD/T is complex and multifaceted, further feeding into a racialized narrative that fosters suffering through this process alone, exacerbating isolation [21]. It may be helpful for people with SCD to develop a reproductive life plan earlier, as this can support mental health and encourage communication in their close relationships. Creating a reproductive life plan allows individuals to make informed decisions about family planning, considering the potential genetic implications of SCD [22]. This proactive approach can help reduce anxiety and uncertainty, fostering a sense of control and preparedness. Additionally, discussing reproductive goals and options with healthcare providers, such as genetic counselors and reproductive endocrinologists, can provide valuable insights and support. These professionals can guide individuals through various family planning options, including preimplantation genetic testing (PGT) and in vitro fertilization (IVF), which can help reduce the risk of having a child with SCD. Engaging in open conversations with partners and family members about reproductive plans can strengthen relationships and ensure that everyone is on the same page [22]. This communication can also help their partners understand the emotional and physical challenges associated with SCD, leading to increased empathy and support that is required even more in the relationship if the couple elects to have a child.

Dating is an important part of a person’s life course, but SCD disrupts that trajectory [21]. Individuals with SCD often face unique challenges in their dating lives, including managing the physical symptoms of the disease, such as pain crises and fatigue, which can limit social activities and spontaneity. These symptoms can make it difficult to maintain a consistent social life, impacting their opportunities to meet potential partners. Moreover, the emotional and psychological burden of living with a chronic illness can affect self-esteem and confidence, making it harder to initiate and sustain romantic relationships. Concerns about how and when to disclose their condition to a potential partner can also add stress and anxiety to the dating process.

The need for regular medical appointments and potential hospitalizations can further complicate dating, as it requires understanding and flexibility from partners. This can sometimes lead to feelings of isolation or fear of being a burden, which may discourage individuals with SCD from pursuing romantic relationships. Despite these challenges, many individuals with SCD desire and successfully maintain fulfilling romantic relationships. Support from healthcare providers, counselors, and support groups can play a crucial role in helping individuals with SCD navigate the complexities of romantic relationships. Open communication with partners about the realities of living with SCD can foster understanding and strengthen their bond.

Overall, developing a reproductive life plan is a crucial step for individuals with SCD, promoting mental well-being, informed decision-making, and stronger interpersonal connections. In addition, while SCD can disrupt the typical dating trajectory, with the right support and strategies, individuals with SCD can build meaningful and lasting romantic connections.

### 4.2. Discussion on the SCD/T Knowledge

The second theme, “SCD/T Knowledge”, emphasizes the role of understanding SCD/T and its genetic implications for childbearing. The participants’ experiences reveal a significant gap in knowledge about SCD/T-specific reproductive health. Many women expressed difficulties in accessing accurate information and genetic counseling, which are crucial for making informed decisions about their reproductive futures. The lack of comprehensive knowledge often leads to uncertainty and anxiety, further complicating their romantic relationships. Despite having a basic understanding of SCD/T, most participants were not well informed about the specific reproductive health issues related to their condition, highlighting a critical area for intervention and education.

The understanding of how SCD affects sexual and reproductive health is generally extremely limited, though at the same time it is well documented that the desire for parenthood for this group of young persons is high [20], not surprisingly, as it is one of the age-appropriate tasks young adults will wrestle with. This gap in knowledge can lead to significant challenges for individuals with SCD who wish to start a family. A limited understanding of the impact of SCD on sexual and reproductive health can result in inadequate preparation and support for managing potential complications during pregnancy and childbirth. This lack of information can also contribute to increased anxiety and uncertainty about reproductive choices.

Educational initiatives and targeted counseling are essential to bridge this knowledge gap, providing patients with the information they need to make informed decisions about their reproductive health. These initiatives can include workshops, informational materials, and one-on-one counseling sessions with healthcare providers who specialize in sickle cell and reproductive health. By improving reproductive health education and awareness, healthcare providers can help patients with SCD/T navigate their reproductive journeys with greater confidence and support. This approach not only addresses the physical aspects of reproductive health but also promotes mental and emotional well-being, ultimately leading to better health outcomes for both parents and children.

### 4.3. Discussion on the Mental and Emotional Toll of SCD/T

The third theme, the “Mental and Emotional Toll of SCD/T”, delves into the psychological challenges and emotional burdens associated with living with SCD/T. The chronic and unpredictable nature of the disease significantly impacts the mental health of affected women, influencing their choices of romantic partners and their coping mechanisms. The participants reported experiencing mental health concerns such as depression, stress, isolation, stigma, and fatalism. The psychosocial challenges of navigating young adult life with SCD/T, particularly in the context of dating, are immense. The constant uncertainty regarding their reproductive future and the fear of passing on the disease to their offspring weigh heavily on their minds. Some participants expressed a preference to avoid romantic relationships and having children altogether, rather than risk the potential suffering of their future children, a stance most often supported and possibly even “pushed” on them by potentially even well-meaning providers [21,22,23,24].

The combination of reproductive injustices, reproductive health, and SCD/T significantly impact individuals’ overall mental and emotional health. Pregnant individuals with SCD/T face a much higher risk of maternal mortality compared to those unaffected [23]. Historically, and possibly in recent times, individuals with SCD have been pressured or coerced into sterilization procedures, and this undoubtably creates feelings of a loss of reproductive autonomy, which can then impact mental health [12,24]. This is outside of the discrimination and bias that people of color already face within the healthcare system. All of these, coupled with the chronic pain that is experienced due to SCD, the social isolation, the stigma attached to the disease, and the fear of passing on the disease to a child, compound the emotional and mental health concerns. Addressing these injustices to therefore support mental and emotional health should take a multifaceted approach, including better education, improved access to reproductive health services, and efforts to eliminate bias and discrimination in healthcare. One issue that came up several times was the relative unpreparedness of providers to help women navigate this. This was on top of a paucity of providers for SCD/T, with few, if any, OBGYN and/or fertility specialists available to the average person with SCD/T. We therefore suggest providers’ training to include this important issue that sadly few are prepared to tackle.

The themes identified in this study highlight the intricate relationship between reproductive health concerns and mental health among women with SCD/T. The findings underscore the need for comprehensive support systems, including genetic counseling and mental health services, and basic fertility provider training to help these women navigate the complex decisions related to their reproductive health and romantic relationships. To effectively implement such programs, healthcare institutions should integrate genetic counseling into routine care for individuals with SCD/T, ensuring early discussions about reproductive options. This could involve multidisciplinary teams, including hematologists, obstetricians, genetic counselors, and mental health professionals, to provide personalized guidance [2]. Addressing these issues is crucial for improving the overall well-being and quality of life for women living with SCD/T.

One major takeaway from this current research is that there is a need for reproductive health education and counseling for patients with SCD/T. This is well aligned with the previous literature suggesting that it should be integrated into standard SCD care [2,18]. In particular, addressing disparities in awareness and healthcare access for minority populations—similar to the challenges observed in blood donation participation—may improve reproductive health outcomes in SCD patients [25,26]. This integration would ensure that patients receive comprehensive care that addresses not only their physical health but also their reproductive and mental well-being. Reproductive health education can empower patients with SCD/T to make informed decisions about family planning, contraception, and pregnancy management. This is particularly important given the potential complications associated with SCD/T during pregnancy, such as an increased risk of pain crises, preterm labor, and other maternal and fetal complications. Counseling can provide a supportive environment for patients to discuss their reproductive goals and concerns, helping to alleviate anxiety and improve their overall quality of life. It can also facilitate discussions about genetic risks and the options available for reducing the likelihood of passing SCD/T to offspring, such as preimplantation genetic testing (PGT) and in vitro fertilization (IVF). By integrating reproductive health education and counseling into standard SCD care, healthcare providers can offer a more holistic approach that addresses the unique needs of patients with SCD/T. This approach not only supports physical health but also promotes mental and emotional well-being, ultimately leading to better health outcomes.

This study has several limitations. First, while the purposive triangulated sampling ensured a broad representation of perspectives, the findings may not be generalizable beyond the study population. The sample size (*N* = 54) was appropriate for qualitative research, but larger studies may further explore the diversity of reproductive health concerns among individuals with SCD/T. Second, the study relied on self-reported experiences, which may be subject to recall bias or social desirability bias. Finally, the study sample consisted of patients, caregivers, advocates, and healthcare providers, but additional perspectives from other stakeholders, such as the partners of individuals with SCD/T, may provide further insights into the reproductive health challenges and support needs of this population.

## 5. Conclusions

Reproductive concerns and experiences among women with SCD/T influence their mental health and social engagement. Programs are urgently needed that address the unique SCD/T reproductive health risks, as well as communication and support needs, for patients and their partners as well as their providers. This includes readily accessible, age-appropriate SCD/T-specific reproductive health information and engaging communication tools with support delivered in a multidisciplinary approach.

## Figures and Tables

**Table 1:** Odds ratios for complications of sickle cell disease that increase maternal and fetal health risks.

ODDS RATIOS FOR COMPLICATIONS OF SICKLE CELL DISEASE THAT INCREASE MATERNAL AND FETAL HEALTH RISKS			
	Cardiomyopathy	3.7	
	Pulmonary HTN	6.3	
	Renal failure	3.5-5.19	
	Anemia	90.1	
	Substance abuse	1	
**REPRODUCTIVE COMPLICATIONS WITH SICKLE CELL DISEASE**			
**Maternal Complications**	**OR**	**Perinatal Complications**	**OR**
Cerebrovascular event	2.0–10.9	Gestational HTN and preeclampsia	1.2–2.7
Deep vein thrombosis	2.5–13.6	Eclampsia	2.7–3.2
Transfusions	22.5	Abruption	1.6
Postpartum hemorrhage	0.5–2.3	Antepartum bleeding	1.7
Pulmonary embolism	1.7–14.35	Preterm labor, preterm delivery	1.1–2.2
Pneumonia	9.8	Intrauterine growth restriction	2.2–4.0
Pyelonephritis	1.3	Intrauterine fetal death (stillbirth)	1.1–26.8
Postpartum infection	1.4	Gestational diabetes mellitus	0.7–1.0
Sepsis	6.8–14.9	Genitourinary tract infection	2.3–6.8
Systemic inflammatory response syndrome	4.1–12.6		
Acute respiratory distress syndrome, mechanical ventilation	6.3–12.2		
Hysterectomy	2.3		

Note: Compared to women without SCD, data from two U.S. national databases, a California Department of Public Health database, and a systematic review [6,7,8,9]. HTN stands for hypertension while OR stands for odds ratios.

## Data Availability

The data presented in this study are available on request from the corresponding author to maintain the privacy of the participants.
